# Supplementary material for: Solar-Driven Syngas Production Using Al-Doped ZnTe Nanorod Photocathodes
Source: Materials (Basel). 2022 Apr 25;15(9):3102. doi: 10.3390/ma15093102 (PMC9103245; doi:10.3390/ma15093102)
Supplement: Supplementary file 1 [file materials-15-03102-s001.zip › materials-1683727-supplementary.pdf]

materials

MDPI

Supplementary Materials

Solar-Driven Syngas Production Using Al-Doped ZnTe Nanorod Photocathodes

Youn Jeong Jang *, Chohee Lee, Yong Hyun Moon and Seokwoo Choe

Department of Chemical Engineering, Hanyang University, Seongdong-gu, Seoul 04763, Korea; chgml9612@hanyang.ac.kr (C.L.); myh4791@hanyang.ac.kr (Y.H.M.); choe5192@hanyang.ac.kr (S.C.)
* Correspondence: yjang53@hanyang.ac.kr; Tel.: +82-2-2220-0526

Citation: Jang, Y.J.; Lee, C.; Moon, Y.H.; Choe, S. Solar-Driven Syngas Production Using Al-Doped ZnTe Nanorod Photocathodes. *Materials* **2022**, *15*, 3102. https://doi.org/10.3390/ma15093102

Academic Editor: Leszek Zaraska

Received: 31 March 2022
Accepted: 21 April 2022
Published: 25 April 2022

Publisher's Note: MDPI stays neutral with regard to jurisdictional claims in published maps and institutional affiliations.

Copyright: © 2022 by the authors. Licensee MDPI, Basel, Switzerland. This article is an open access article distributed under the terms and conditions of the Creative Commons Attribution (CC BY) license (https://creativecommons.org/licenses/by/4.0/).

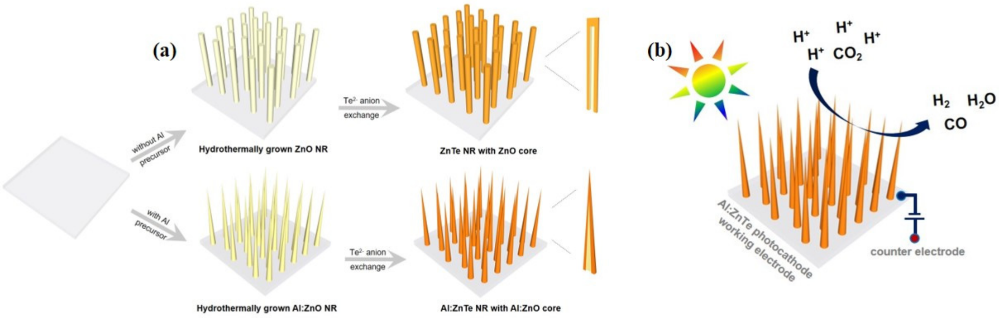

Scheme S1. Schematic diagram for (**a**) ZnTe NR and Al:ZnTe NR film synthesis and (**b**) photoelectrochemical syngas production set-up.

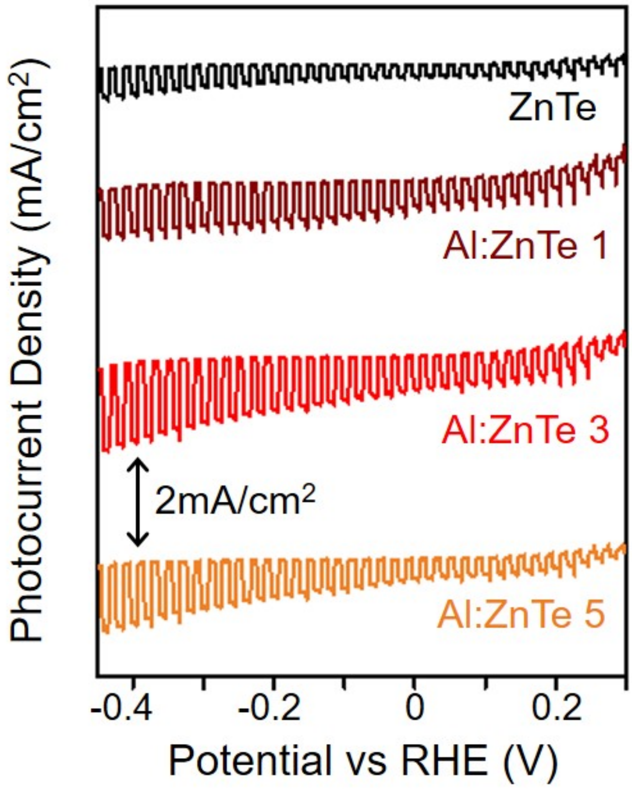

Figure S1. J-V curves for ZnTe and Al:ZnTe X photocathodes where X indicates molar concentration of Al dopants of 1, 3, and 5 %. All PEC measurements were performed in a CO_2 saturated $KHCO_3$ under chopped simulated solar light irradiation (AM 1.5G, 100mW/cm^2).

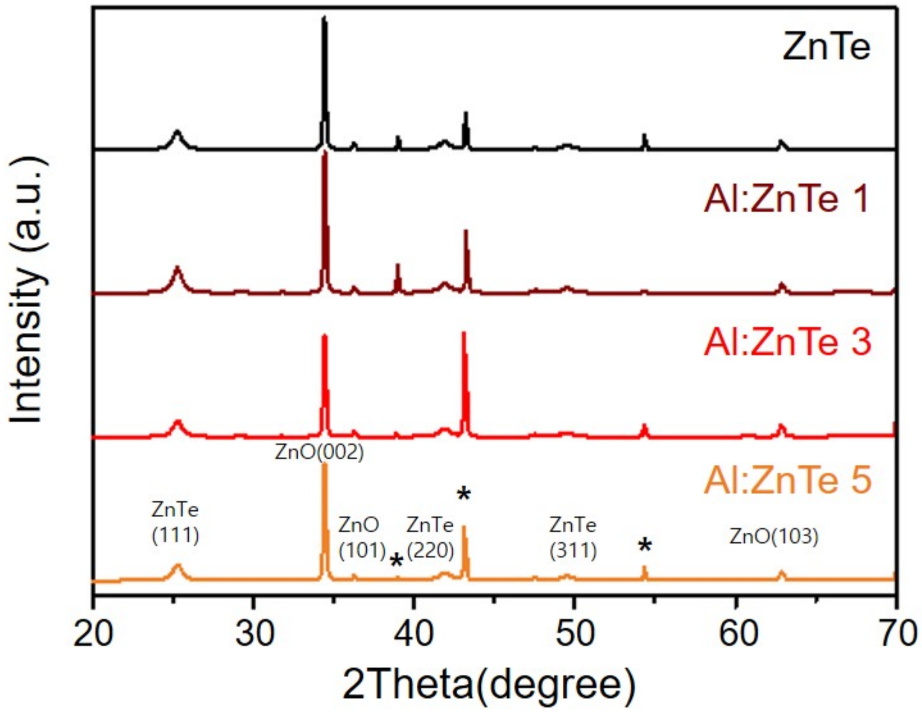

Figure S2. XRD patterns for ZnTe and Al:ZnTe X, where X indicates molar concentration of Al dopants of 1, 3, and 5 %. The symbol of * indicates XRD patterns for FTO substrate

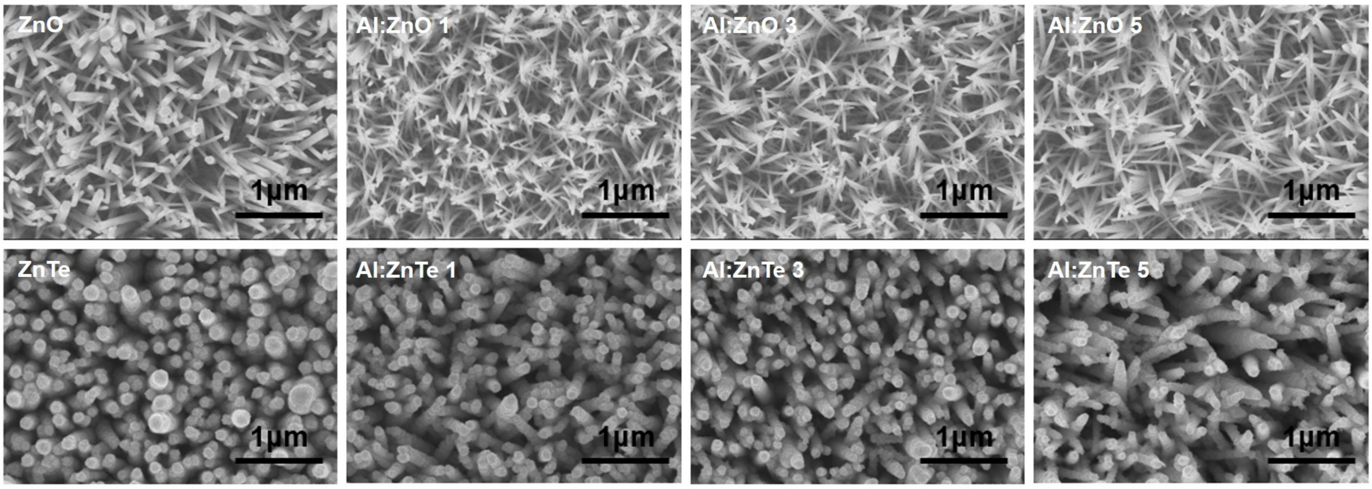

Figure S3. SEM images for ZnO, ZnTe, Al:ZnO X and Al:ZnTe X, where X indicates molar concentration of Al dopants of 1, 3, and 5 %.
